# The diagnosis of aspiration pneumonia in older persons: a systematic review

**DOI:** 10.1007/s41999-022-00689-3

**Published:** 2022-08-25

**Authors:** Yuki Yoshimatsu, Dorte Melgaard, Albert Westergren, Conni Skrubbeltrang, David G. Smithard

**Affiliations:** 1 Elderly Care, Queen Elizabeth Hospital, Lewisham and Greenwich NHS Trust, Stadium Rd, London, SE18 4QH UK; 2 Centre for Exercise Activity and Rehabilitation, School of Human Sciences, University of Greenwich, London, UK; 3 Centre for Clinical Research, North Denmark Regional Hospital, Hjoerring, Denmark; 4 Department of Clinical Medicine, Aalborg University, Aalborg, Denmark; 5 The Research Platform for Collaboration for Health, Faculty of Health Sciences, Kristianstad University, Kristianstad, Sweden; 6 Medical Library, Aalborg University Hospital, Aalborg, Denmark

**Keywords:** Dysphagia, Aspiration, Pneumonia, Diagnosis, Geriatric, Swallowing disorders

## Abstract

**Purpose:**

Community-acquired pneumonia (CAP) is highly common across the world. It is reported that over 90% of CAP in older adults may be due to aspiration. However, the diagnostic criteria for aspiration pneumonia (AP) have not been widely agreed. Is there a consensus on how to diagnose AP? What are the clinical features of patients being diagnosed with AP? We conducted a systematic review to answer these questions.

**Methods:**

We performed a literature search in MEDLINE^®^, EMBASE, CINHAL, and Cochrane to review the steps taken toward diagnosing AP. Search terms for “aspiration pneumonia” and “aged” were used. Inclusion criteria were: original research, community-acquired AP, age ≥ 75 years old, acute hospital admission.

**Results:**

A total of 10,716 reports were found. Following the removal of duplicates, 7601 were screened, 95 underwent full-text review, and 9 reports were included in the final analysis. Pneumonia was diagnosed using a combination of symptoms, inflammatory markers, and chest imaging findings in most studies. AP was defined as pneumonia with some relation to aspiration or dysphagia. Aspiration was inferred if there was witnessed or prior presumed aspiration, episodes of coughing on food or liquids, relevant underlying conditions, abnormalities on videofluoroscopy or water swallow test, and gravity-dependent distribution of shadows on chest imaging. Patients with AP were older, more frailer, and had more comorbidities than in non-AP.

**Conclusion:**

There is a broad consensus on the clinical criteria to diagnose AP. It is a presumptive diagnosis with regards to patients’ general frailty rather than in relation to swallowing function itself.

**Supplementary Information:**

The online version contains supplementary material available at 10.1007/s41999-022-00689-3.

## Key summary points

**Aim:**

A systematic review was performed to identify how aspiration pneumonia is being diagnosed in older persons, as there is no definitive criteria to differentiate it from non-AP.

**Findings:**

There is a broad consensus on the diagnostic criteria of AP, consisting of pneumonia in the context of presumed aspiration or documented dysphagia in the presence of increasing age and frailty.

**Message:**

Currently, AP may be a presumptive diagnosis with regards to the patient’s general frailty rather than in relation to swallowing function itself.

**Supplementary Information:**

The online version contains supplementary material available at 10.1007/s41999-022-00689-3.

## Introduction

The estimated prevalence of community-acquired pneumonia (CAP) in the world is between 150 and 1400/100 000 [1]. The mortality rate of CAP is 2–5/1000 years [2, 3] and 1.13 million or 261/100 000 people > 70 years of age died secondary to CAP in 2017, a 9% increase in mortality of people over the previous 3 decades [4].

Many people consider CAP in the older population to be secondary to AP [5]. However, the diagnostic criteria of aspiration pneumonia (AP) are unclear and definitions are frequently inconsistent [6]. The British Thoracic Society does not have any guidance for the definition or management of pneumonia [7], nor does the American Thoracic Society [8]. The Japanese Respiratory Society defines AP as “pneumonia occurring in the context of dysphagia and risk of pneumonia” [9], taking in the fact that dysphagia and aspiration on their own does not necessarily result in infection [10]. The most recent BMJ best practice guidance defines aspiration pneumonia as inhalation of oropharyngeal contents into the lower airways leading to chemical pneumonitis and thence bacterial pneumonia [11]. It might, however, be difficult to establish a clear causal relationship between aspiration and pneumonia, due to the time gap between one or several aspiration occasions and the development of pneumonia.

As the definition in unclear, it is common for frail older adults to be diagnosed presumptively with aspiration pneumonia [12, 13]. It has been suggested that the prevalence may be as high as 90% among older patients hospitalized with CAP [5, 6, 14, 15]. The inference to be drawn from the literature is that anyone who is frail or may have evidence of a swallowing problem and develops a CAP most likely has an aspiration [12].

Many older adults with a clinical diagnosis of pneumonia will have underlying swallowing problems, also known as presbyphagia [16]. Saliva regularly enters the bronchial tree, and the oropharynx and lungs have a similar microbiome; then how do clinicians differentiate between CAP and AP in frail older adults? Authors have questioned whether AP exists as a distinct clinical entity [17].

Taking these clinical controversies into account, we have conducted a systematic review of the literature to identify those clinical patient features which are taken to indicate a diagnosis of AP rather than CAP.

## Methods

### Study design

A systematic review, following PRISMA guidelines [18], was conducted of the scientific literature reporting studies of the diagnosis and of AP in the older adult population.

### Search strategy

The following databases were searched: Ovid MEDLINE^®^, Ovid EMBASE, CINHAL with full text from Ebsco, and Cochrane Library. All databases were searched on July 21st, 2021.

The search strategy was developed by a librarian (CS) in cooperation with the other authors. The search strategy was developed in Medline and subsequently translated into other databases. We searched for “aspiration pneumonia” or “deglutition disorders and pneumonia” and “aged” using both controlled vocabularies such as MeSH terms and natural language terms for their synonyms. We excluded guidelines, meta-analyses, reviews and case reports. The search was limited to articles in English, Danish, Swedish, Norwegian, German and Japanese.

A total of 7601 unique citations were found. Duplicates were removed using Endnote and Covidence^®^ duplicate identification strategies.

The search strategies for each database are listed in the supplementary information.

### Study selection

Identified studies were reviewed by two of the authors (YY and DGS) independently and decisions were recorded via Covidence^®^. Where agreement was not reached, papers were reviewed by two other authors (DM and AW). Duplicate papers/reports were removed prior to screening.

Inclusion terms/criteria included original papers, CAP, geriatric population, 75 years and older, and hospital. Exclusion terms/criteria were: reviews, case reports, editorials, conference papers, studies with mixed populations, nursing home residents, non-acute environment (rehabilitation centers), post-operative/ post-endoscopic aspiration pneumonia, COVID-19 related pneumonia, hospital-acquired pneumonia, ventilator-assisted pneumonia, and stroke (Table 1). Manual searches were also performed from the reference list of included studies.

**Table 1:** Inclusion and exclusion criteria

Inclusion criteria	Exclusion criteria
Original studies on aspiration pneumonia	Reviews
Community acquired pneumonia	Case reports
≥ 75 years old	Editorials
Acute hospital	Conference papers
In English, Swedish, Danish, Norwegian, German, or Japanese	Mixed populations
	Nursing home
	Non-acute environment
	Post-operative or post-endoscopic pneumonia
	COVID-19 related pneumonia
	Hospital-acquired pneumonia
	Ventilator-associated pneumonia
	Stroke related pneumonia
	In languages other than listed in the inclusion criteria

### Data collection

A data extraction form was designed to extract study characteristics and diagnostic measures taken regarding AP. YY and DGS collected data from eligible publications independently. Extracted data were compared, and any discrepancies were resolved through discussion. No automation tools were used. As this review was not intended to find outcomes but rather a descriptive study to identify those factors that would lead to aspiration pneumonia, we extracted information relating to the characteristics of included studies and results as follows: author, year, source of publication, sample size, sample/participant characteristics, diagnosis of pneumonia, diagnosis of aspiration/dysphagia, and conclusion (Table 2). The quality of the studies was evaluated according to the Newcastle–Ottawa Scale (NOS) [19]. However, as the purpose of our review was to focus on the diagnosis of pneumonia and aspiration rather than the outcomes of each study, the latter five items of the NOS were not relevant. Therefore, we rated the studies based on the first three items of the NOS, namely, the representativeness of the exposed cohort, the selection of the non-exposed cohort, and the ascertainment of the exposure (Table 3).

**Table 2:** Characteristics of included studies

No.	Author country, year	Study design	Objectives	Participants	Participants/controls	Diagnosis of pneumonia	Diagnosis of aspiration/dysphagia	Conclusion
1	Katsura [20]. Japan, 1998	Retrospective	Outcomes in patients with recurrent pulmonary aspiration	Hospitalized older persons with repeated aspiration events (≥ 1/week)	38/0	Symptoms (fever, cough, sputum), inflammatory markers	Witnessed aspiration during eating and requiring intervention such as suction	1. Repeated aspiration mostly occurs with underlying diseases of CVD, dementia, and deterioration of ADLs; 2. Prognosis is poor; PEG contributes to survival but does not prevent pneumonia
2	Tokuyasu [21]. Japan, 2009	Prospective cohort	1. Causative organisms of AP (bronchoscopy), 2. Efficacy of meropenem for AP	Hospitalized patients (≥ 75 yo) with AP	62/0	Symptoms (fever, cough, purulent sputum), blood tests, lung infiltration on X-ray and CT	1:aspirated content detected in respiratory tract, 2:coughing or choking before/during/or after swallowing, and 3:dysphaagia on videofluoroscopy	1. Anaerobic bacteria coverage may be necessary for potentially fatal AP; 2.Meropenem is effective and tolerable
3	Takenaka [22]. Japan, 2011	Prospective cohort	Factors related to repetitive AP in older persons with dysphagia	Admissions for AP ≥ 2 times during study period (control: admitted once)	15/53	Symptoms suggesting respiratory infection, inflammatory markers, and X-ray	Symptoms suggesting aspiration prior to admission	The relapse group had higher rates of coming from institutions or hospitals, and higher brain dysfunction
4	Bosch [23]. Spain, 2012	Prospective cohort	Mortality rate and prognostic factors in old patients with dementia, hospitalized for AP	≥ 75 yo admissions with AP with dementia	120/0	Chest infiltration and 1 major criteria (cough, sputum, BT ≥ 37.8°) or 2 minor criteria (dyspnoea, pleuritic pain, delirium, RR > 20, consolidation, WBC > 12,000/µL)	Risk factors for oropharyngeal aspiration and a history of witness or suspected aspiration	In-hospital and 6 month mortality were high (33.3%, 50.8%). Multilobar involvement and lower lymphocyte counts were associated with hospital mortality, and older age, greater dependence and malnutrition with 6 month mortality
5	Komiya [24]. Japan, 2013	Retrospective	CT features of AP	Admissions for pneumonia who were subsequently confirmed to have dysphagia by VF	53/0	Not mentioned	On VF: disability to move food or liquid from the mouth through the pharynx and oesophagus into the stomach safely and efficiently	Common patterns were bronchopneumonia and bronchiolitis pattern. Distribution was characterized by gravity dependence
6	Pinargote [25]. Spain, 2015	Prospective cohort	Clinical features and outcomes of AP and non-AP	≥ 80 yo admitted with AP (control: non-AP)	46/30	Radiographic evidence of pulmonary infiltration and acute onset of symptoms of LRTI	Infiltration in posterior segments of upper lobes or apical/basal segments of lower lobes and vomiting or witnessed aspiration, or risks for aspiration (dementia, CVD, NMD, pharyngolaryngeal dysfunction, oesophageal dysfunction or mechanical obstruction, tube feeding, gastroesophageal reflux, or poor swallowing previously confirmed)	AP showed higher levels of sodium, low estimated glomerular filtrate rate, higher severity of pneumonia, and slightly higher mortality than non-AP
7	Palacios-Cena [26]. Spain, 2017	Retrospective	1. AP hospitalizations according to sex and comorbidities, 2. Time trends in outcomes, 3. Factors associated with in-hospital mortality	≥ 75 yo admitted and with a primary diagnosis of AP according to ICD-9-CM (using national database)	111,319/0	Not mentioned	AP event codes according to the ICD-9-CM: 507.x (pneumonitis or pneumonia caused by inhalation of vomitus or food)	AP patients were older, more male, and had more comorbidities. Over time, length of hospital stay and in-hospital mortality decreased in both sexes, but readmissions increased significantly in females
8	Nakashima [27]. Japan, 2018	Prospective cohort	Association of silent aspiration and mortality in AP	≥ 65 yo admitted for AP (2 acute hospitals, Japan)	170/0	New gravity-dependent shadow on chest X-ray/CT, and ≥ 2 of the following: BT ≥ 37.5 °C, high CRP, WBC ≥ 9000/µL, purulent sputum	Positive water swallowing test or condition related to aspiration (neurological disorder, bedridden, severe cognitive impairment or gastroesophageal reflux)	Silent aspiration detected on cough latency test can predict 1-month mortality in older AP
9	Manabe [28]. Japan, 2020	Retrospective	Factors to distinguish AP from CAP in primary care	AP in primary care database of 20 hospitals and clinics (control: CAP)	130/58	Not mentioned	Overall clinical assessment, risk factors for aspiration, and/or chest radiograph abnormalities	Characteristic factors for diagnosing AP in the oldest-old in primary care settings are: nursing home and dysphagia risks (cerebral infarction, dementia, hypertension)

*yo* years-old, *CVD* cerebrovascular disease, *ADL* activities of daily life, *PEG* percutaneous endoscopic gastrostomy, *AP* aspiration pneumonia, *BT* body temperature, *RR* respiratory rate, *WBC* white blood cells, *CT* computed tomography, *VF* videofluoroscopy, *NMD* neuromuscular disease, *CAP* community-acquired pneumonia

**Table 3:** Quality assessment of included studies

No	Author country, year	Representativeness of patient cases	Selection of the non-exposed cohort	Ascertainment of exposure	Total number of stars (1–3)
1	Katsura [20]. Japan, 1998	b	c	d	1
2	Tokuyasu [21]. Japan, 2009	a	c	a	2
3	Takenaka [22]. Japan, 2011	b	a	a	3
4	Bosch [23]. Spain, 2012	a	c	a	2
5	Komiya [24]. Japan, 2013	a	c	d	1
6	Pinargote [25]. Spain, 2015	a	a	a	3
7	Palacios-Cena [26]. Spain, 2017	a	c	d	1
8	Nakashima [27]. Japan, 2018	a	a	a	3
9	Manabe [28]. Japan, 2020	a	a	a	3

Assessment scores: Representativeness of the exposed cohort: (a) truly representative of the average older persons’ pneumonia in the community, (b) somewhat representative of the average older persons’ pneumonia in the community, (c) selected group, (d) no description of the derivation of the cohort. Selection of the non-exposed cohort: (a) drawn from the same community as the exposed cohort, (b) drawn from a different source, (c) no description of the derivation of the non-exposed cohort. Ascertainment of exposure: (a) secure record, (b) structured interview, (c) written self-report, (d) no description

## Results

**Fig. 1:**
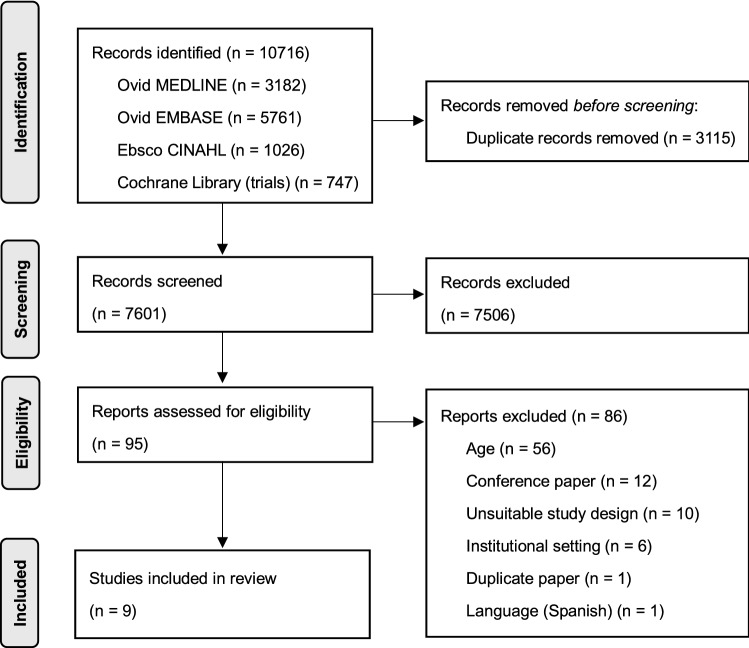
Flow chart of the study process. Through searching databases, 10 716 reports were found. After removing duplicates, 7601 reports were screened, of which 7506 were excluded. A total of 95 studies underwent full-text review, and 9 studies were included in the review

Upon studying databases and a manual search, 10,716 reports were found. 3115 duplicate reports were removed. The remaining 7601 reports were screened on their titles and abstracts, of which 7506 were excluded (Fig. 1). Among the 95 studies undergoing full-text review, 86 were excluded due to the following reasons: the age group did not meet the criteria (*n* = 56), it was a conference paper (*n* = 12), the study design was unsuitable (*n* = 10), the institutional setting (*n* = 6), duplicate paper (*n* = 1), and the language being Spanish (*n* = 1). As for the ten studies which were excluded for the design being unsuitable, eight were not focused on aspiration pneumonia, and two were reviews. Therefore, a total of nine articles were included in the final analysis [20–28]. The study selection process is illustrated in Fig. 1, according to the PRISMA methodology [18]. Two studies were found to be relevant from manual searches amongst the list of references in the 95 studies undergoing full-text review; both were excluded due to one being a review [29], and another being a younger age group [14].

Of the nine included studies, there were five prospective cohort studies [21–23, 25, 27] and four retrospective studies [20, 24, 26, 28]. Six of these studies were from Japan [20–22, 24, 27, 28], and three were from Spain [23, 25, 26]. Overall, 112 094 patients were involved in all studies.

### Diagnosis of pneumonia

Most studies used a combination of symptoms (fever, cough, sputum), raised inflammatory markers, and chest imaging findings [20–23, 25, 27], although three studies did not mention the criteria [24, 26, 28]. One of these was a study performed using the national database [26].

### Aspiration or dysphagia diagnosis

All studies considered AP as pneumonia with some sort of factor related to aspiration or dysphagia. All studies had varied combinations of witnessed aspiration, episodes of coughing on food or liquids [20, 21, 23, 25], underlying conditions [23, 25, 27, 28], assessments (video fluoroscopy or water swallow test) [21, 24, 27], and gravity-dependent distribution of shadows on chest imaging (CT or radiograph) [25, 27, 28]. One study specified that there must be an aspiration witnessed by a doctor or nurse followed by an intervention such as suctioning [20], four accepted a witnessed aspiration or history of coughing on food or other risk factors, or an abnormal assessment result [21, 25, 27, 28]. One study relied solely on prior symptoms of aspiration [22], while another had video fluoroscopy as their sole criteria [24].

### Characteristics of patients with aspiration pneumonia

Those diagnosed with aspiration pneumonia were older, more likely to have malnutrition [23] had a high rate of common medical comorbidities of frailty including cerebrovascular disease (15.8–80.0%) [20–25, 27], dementia (34.9–93.3%) [20–25, 27], and being bedridden (38.0–97.4%) [20–22, 24]. Patients with these characteristics were likely to have recurrent pneumonia and have a worse prognosis [20, 22, 26].

### Study quality

There was variability in study quality (Table 3). Four studies scored the maximum number of stars [22, 25, 27, 28]. All studies scored the first item, namely, the representativeness of the exposed cohort. Six did not score on the ‘selection of the non-exposed cohort’ as they did not have a control group [20, 21, 23, 24, 26], and three only scored one star due to not meeting the ‘ascertainment of exposure’ [20, 24, 26].

## Discussion

We have conducted a systematic review, following the principles of the PRISMA guidelines, of the steps taken towards diagnosing AP in non-institutionalized older adults aged ≥ 75 years admitted to hospital acutely.

### Diagnosis of aspiration pneumonia

Of the nine studies included, the methodology used to diagnose pneumonia and aspiration varied. The consensus was that AP was defined as pneumonia with some sort of factor related to aspiration or dysphagia. Aspiration was inferred if there was witnessed or history of prior aspiration, episodes of coughing on food or liquids, relevant underlying conditions, videofluoroscopy or water swallow test, and gravity-dependent distribution of shadows on chest imaging.

In many countries, there are no clear coherent diagnostic criteria or definition of aspiration pneumonia, and as consequence there is variability in the clinical identification of aspiration pneumonia and the subsequent clinical management [7, 8, 11].

In clinical practice, a diagnosis of pneumonia is generally established from the presence of a combination of symptoms, inflammatory markers, and radiographic changes [7–9]. A diagnosis of AP is inferred if pneumonia occurs in the presence of documented dysphagia. There is no consensus on the definition of dysphagia and clinicians use a variety of criteria among the following: history of risk factors, history of coughing on food, history of recurrent pneumonia, witnessed coughing on food, suctioning of aspirated material from the airway, gravity-dependent radiographic changes, and swallow screening/assessment outcomes [20–28]. Depending on the environment or clinician/researcher, the extent to which each of these criteria are considered in the diagnostic process differs widely. However, there remains the possibility that pneumonia is not entirely related to the presence of dysphagia [10, 12, 17].

The results of this review suggest that AP is more likely to be diagnosed in older adults who are frail or suffer from more than one long-term (chronic) medical condition and as a consequence have an impaired immune response, making them susceptible to infection, rather than the presence of dysphagia itself [10]. Therefore, a diagnosis of AP may simply infer that a patient with a previous history of stroke, terminal stages of dementia or neurodegenerative conditions, has developed an incidental CAP, or a patient who happened to cough whilst eating had concurrent pneumonia. On the other hand, a patient who has a background of dementia or stroke but has not been diagnosed may be regarded as CAP when they may have developed AP. In this manner, clinicians’ decisions can be highly biased by past medical histories and records.

The Japanese Respiratory Society has recognized this conundrum and has emphasized in its recommendations that the cause of pneumonia in the presence of dysphagia and possible aspiration may be due to a person’s overall medical and physical condition rather than merely due to dysphagia itself, and their guidance only list the risk of aspiration and pneumonia without stating clear diagnostic criteria [9]. The American and British guidelines do not allocate a section for aspiration pneumonia as an individual entity [7, 8]. Rather they separate pneumonia according to whether the pneumonia was CAP or hospital/healthcare acquired.

The literature has suggested that the underlying aetiology for pneumonia in older people is aspiration and therefore, clinical staff should consider the possibility of aspiration and dysphagia in all pneumonia in the older population, by taking a careful history and dysphagia screening as needed, instead of differentiating between aspiration related and non-aspiration related pneumonia at an early stage [30, 31]. As found in our review, many patients diagnosed with aspiration pneumonia had underlying conditions such as stroke and dementia. Clinically, it has become common to consider aspiration when patients are frail and have multiple co-morbidities present. However, in patients who are not frail or pre-frail and do not have multiple comorbidities, the possibility of aspiration being present may not be considered by clinicians. This highlights the importance of careful history taking and physical examination (including swallowing screening/assessment [30–33], rather than attempting to differentiate between AP and CAP.

Interestingly, all of the included studies were from either Japan or Spain. This is suspected to be due to mainly two reasons. These two countries have constantly ranked among the top in life expectancy in recent years [34]. Social systems concerning the older population may also affect the researchers’ decision of age criteria in their studies. For example, in Japan, there is a specific category for those aged +75 years old called ‘Kouki-koureisha’ or late-stage older persons, as opposed to those aged 66–74 years old (the early-stage older persons). In Spain, it is common for 70 years old to be the cutoff age for patients being considered ‘older’. This may have impacted the studies to have originated from these two countries. Similarly in the UK, people aged ≥ 75 years are considered ‘old’ and ≥ 85 years ‘old-old’. As the life expectancy increases, it is hoped that more studies in these age groups will arise from countries other than Spain and Japan too.

### Strengths and weaknesses of this study

This review focused on older people living at home. Many of the studies that were initially identified focused on care home/nursing home residents, younger populations, mixed populations, stroke-related pneumonia, and were therefore excluded from this review. This resulted in a significant reduction in the number of publications included. However, many of the findings in those publications excluded after full-text review were similar to those included.

Old age has typically been defined as the age at which you retire and take your pension [35]. The scientific literature often accepts old age as commencing at 65 years, whereas in reality the average life span has improved over the years and those who are between 65 and 74 years are increasingly active and non-frail and do not perceive themselves as old, and are not looked after by “geriatric medicine services”. As the focus of the study was the diagnosis of AP in older adults, the authors’ consensus was to accept the age of 75 years as a lower age for old age.

Studies only originated from two countries (Spain and Japan); it is possible that there may be differences compared to other countries, though local data does not support this (Yoshimatsu Y, Smithard DG; in progress). Further research into the diagnostic process and the consequences of these measures would be beneficial. Ultimately, it is hoped that these studies would lead to better management of pneumonia in older people.

In the current literature, the diagnosis of aspiration pneumonia in older persons is highly related to underlying long-term medical conditions and frailty. In clinical practice, it may be more relevant to assess patients on their underlying general medical and physical condition in conjunction with their ability to swallow safely, rather than attempting to differentiate whether they have an AP or CAP.

## Supplementary Information

Below is the link to the electronic supplementary material.

Supplementary file1 (DOCX 23 KB)
