# Supplementary material for: The diagnosis of aspiration pneumonia in older persons: a systematic review
Source: Eur Geriatr Med. 2022 Aug 25;13(5):1071–80. doi: 10.1007/s41999-022-00689-3 (PMC9409622; doi:10.1007/s41999-022-00689-3)
Supplement: Supplementary file 1 — Supplementary file1 (DOCX 23 KB) [file 41999_2022_689_MOESM1_ESM.docx]

Supplementary Information

1. The search strategy for MEDLINE

| # | Query | Results |
| --- | --- | --- |
| 1 | exp Pneumonia, Aspiration/ | 6,135 |
| 2 | ((aspirat* or inhalat* or mendelson* or deglutition* or lipid* or lipoid or oil) adj3 (pneumon* or syndrome*)).ti,ab,kw,kf. | 9,240 |
| 3 | exp Deglutition Disorders/ | 54,792 |
| 4 | dysphagia*.ti,ab,kw,kf. | 30,362 |
| 5 | ((deglutition* or Swallow*) adj3 (disorder* or dysfunct* or problem* or difficult* or disease* or deficit*)).ti,ab,kw,kf. | 8,685 |
| 6 | 3 or 4 or 5 | 74,273 |
| 7 | exp Pneumonia/ | 187,324 |
| 8 | pneumon*.ti,ab,kw,kf. | 211,261 |
| 9 | ((lung* or pulmon* or airway*) adj3 (infection* or inflammat*)).ti,ab,kw,kf. | 69,285 |
| 10 | 7 or 8 or 9 | 385,233 |
| 11 | 6 and 10 | 3,987 |
| 12 | 1 or 2 or 11 | 14,492 |
| 13 | exp Aged/ | 3,275,018 |
| 14 | exp Aging/ | 254,200 |
| 15 | (aged or geriatric* or gerontol* or elder* or ageing or aging or Old or older or senior*).ti,ab,kw,kf. | 2,361,552 |
| 16 | Geriatrics/ | 30,556 |
| 17 | 13 or 14 or 15 or 16 | 5,014,670 |
| 18 | case reports/ | 2,195,568 |
| 19 | (case report* or case stud*).ti. | 323,458 |
| 20 | exp guideline/ or exp meta-analysis/ or "review"/ or "systematic review"/ or (guideline* or review* or "meta anal*" or metanal*).ti. or cochrane.jw. | 3,229,669 |
| 21 | 18 or 19 or 20 | 5,332,695 |
| 22 | 12 and 17 | 5,567 |
| 23 | 22 not 21 | 3,392 |
| 24 | limit 23 to (danish or english or german or japanese or norwegian or swedish) | 3,182 |

2. The search strategy for Ovid EMBASE

| # | Query | Results |
| --- | --- | --- |
| 1 | aspiration pneumonia/ | 15,630 |
| 2 | ((aspiration* or inhalation* or mendelson* or deglutition* or lipid* or lipoid or oil) adj3 (pneumon* or syndrome*)).ti,ab,kw,hw. | 22,494 |
| 3 | dysphagia/ | 75,264 |
| 4 | ((deglutition* or Swallow*) adj3 (disorder* or dysfunct* or problem* or difficult* or disease* or deficit*)).ti,ab,kw,hw. | 14,677 |
| 5 | dysphagia*.ti,ab,kw,kw. | 52,537 |
| 6 | 3 or 4 or 5 | 90,511 |
| 7 | exp pneumonia/ | 333,365 |
| 8 | pneumon*.ti,ab,kw,hw. | 459,678 |
| 9 | ((lung* or pulmon* or airway*) adj3 (infection* or inflammat*)).ti,ab,hw. | 112,960 |
| 10 | 7 or 8 or 9 | 566,632 |
| 11 | 6 and 10 | 8,794 |
| 12 | 1 or 2 or 11 | 27,318 |
| 13 | exp aged/ | 3,187,915 |
| 14 | exp aging/ | 281,017 |
| 15 | exp geriatrics/ | 38,560 |
| 16 | gerontology/ | 3,179 |
| 17 | (aged or geriatric* or gerontol*or elder* or ageing or aging or Old or senior*).ti,ab,kw,hw. | 6,296,121 |
| 18 | 13 or 14 or 15 or 16 or 17 | 6,303,016 |
| 19 | conference abstract/ | 1,259,124 |
| 20 | exp case study/ | 79,723 |
| 21 | case report/ | 2,631,378 |
| 22 | (case report* or case stud*).ti. | 394,727 |
| 23 | exp "review"/ or exp meta analysis/ or exp practice guideline/ or (guideline* or review* or "meta anal*" or metanal*).ti. or cochrane.jw. | 3,661,511 |
| 24 | 19 or 20 or 21 or 22 or 23 | 7,253,010 |
| 25 | 12 and 18 | 11,900 |
| 26 | 25 not 24 | 6,017 |
| 27 | limit 26 to (danish or english or german or japanese or norwegian or swedish) | 5,761 |

3. The search strategy for CINAHL

| # | Query | Limiters/Expanders | Results |
| --- | --- | --- | --- |
| S24 | s18 NOT s22 | Limiters - Language: Danish, English, German, Japanese, Norwegian, Swedish Search modes - Boolean/Phrase | 1,026 |
| S23 | s18 NOT s22 | Search modes - Boolean/Phrase | 1,043 |
| S22 | S19 OR S20 OR S21 | Search modes - Boolean/Phrase | 1,152,098 |
| S21 | TI (guideline* or review* or "meta anal*" or metanal* OR case stud* OR case report*) | Search modes - Boolean/Phrase | 383,110 |
| S20 | (MH "Practice Guidelines") OR (MH "Case Studies") OR (MH "Literature Review+") OR (MH "Meta Analysis") | Search modes - Boolean/Phrase | 237,177 |
| S19 | PT (Case Study OR Meta Analysis OR Practice Guidelines OR Review OR Systematic Review) | Search modes - Boolean/Phrase | 896,004 |
| S18 | S12 AND S17 | Search modes - Boolean/Phrase | 1,385 |
| S17 | S13 OR S14 OR S15 OR S16 | Search modes - Boolean/Phrase | 1,180,382 |
| S16 | aged or geriatric* or gerontol* or elder* or ageing or aging or Old or senior* | Search modes - Boolean/Phrase | 1,178,121 |
| S15 | (MH "Geriatrics") | Search modes - Boolean/Phrase | 5,738 |
| S14 | (MH "Aging+") | Search modes - Boolean/Phrase | 57,384 |
| S13 | (MH "Aged+") | Search modes - Boolean/Phrase | 876,873 |
| S12 | S1 OR S2 OR S11 | Search modes - Boolean/Phrase | 3,911 |
| S11 | S6 AND S10 | Search modes - Boolean/Phrase | 1,211 |
| S10 | S7 OR S8 OR S9 | Search modes - Boolean/Phrase | 64,737 |
| S9 | ((lung* or pulmon* or airway*) N3 (infection* or inflammat*)) | Search modes - Boolean/Phrase | 9,928 |
| S8 | pneumon* | Search modes - Boolean/Phrase | 56,647 |
| S7 | (MH "Pneumonia+") | Search modes - Boolean/Phrase | 31,929 |
| S6 | S3 OR S4 OR S5 | Search modes - Boolean/Phrase | 14,490 |
| S5 | ((deglutition* or Swallow*) N3 (disorder* or dysfunct* or problem* or difficult* or disease* or deficit*)) | Search modes - Boolean/Phrase | 10,444 |
| S4 | dysphagia* | Search modes - Boolean/Phrase | 9,414 |
| S3 | (MH "Deglutition Disorders") | Search modes - Boolean/Phrase | 9,034 |
| S2 | ((aspiration* or inhalation* or mendelson* or deglutition* or lipid* or lipoid or oil) N3 (pneumon* or syndrome*)). | Search modes - Boolean/Phrase | 3,493 |
| S1 | (MH "Pneumonia, Aspiration") | Search modes - Boolean/Phrase | 1,704 |

4. The search strategy for Cochrane

| ID | Search | Hits |
| --- | --- | --- |
| #1 | MeSH descriptor: [Pneumonia, Aspiration] explode all trees | 336 |
| #2 | ((aspiration* or inhalation* or mendelson* or deglutition* or lipid* or lipoid or oil) NEAR/3 (pneumon* or syndrome*)):ti,ab,kw | 1460 |
| #3 | MeSH descriptor: [Deglutition Disorders] explode all trees | 2957 |
| #4 | (dysphagia*):ti,ab,kw | 4419 |
| #5 | ((deglutition* or Swallow*) NEAR/3 (disorder* or dysfunct* or problem* or difficult* or disease* or deficit*)):ti,ab,kw | 1859 |
| #6 | #3 OR #4 OR #5 | 7178 |
| #7 | MeSH descriptor: [Pneumonia] explode all trees | 4322 |
| #8 | (pneumon*):ti,ab,kw | 20519 |
| #9 | ((lung* or pulmon* or airway*) NEAR/3 (infection* or inflammat*)):ti,ab,kw | 6406 |
| #10 | #7 OR #8 OR #9 | 26135 |
| #11 | #6 AND #10 | 537 |
| #12 | #1 OR #2 OR #11 | 1750 |
| #13 | MeSH descriptor: [Aged] explode all trees | 212777 |
| #14 | MeSH descriptor: [Geriatrics] explode all trees | 207 |
| #15 | (aged or geriatric* or gerontol* or elder* or ageing or aging or Old or senior*):ti,ab,kw | 588720 |
| #16 | #13 OR #14 OR #15 | 588720 |
| #17 | #16 AND #12 | 754 |
|  | Trials | 747 |
